# SynBlast: Assisting the analysis of conserved synteny information

**DOI:** 10.1186/1471-2105-9-351

**Published:** 2008-08-24

**Authors:** Jörg Lehmann, Peter F Stadler, Sonja J Prohaska

**Affiliations:** 1 Bioinformatics Group, Department of Computer Science, University of Leipzig, Härtelstraße 16-18, D-04107 Leipzig, Germany; 2 Interdisciplinary Center for Bioinformatics, University of Leipzig, Härtelstraße 16-18, D-04107 Leipzig, Germany; 3 Fraunhofer Institute for Cell Therapy and Immunology, Perlickstraße 1, D-04103 Leipzig, Germany; 4 Santa Fe Institute, 1399 Hyde Park Rd., Santa Fe, NM 87501, USA; 5 Institute for Theoretical Chemistry, University of Vienna, Währingerstraße 17, A-1090 Wien, Austria; 6 Biomedical Informatics, Arizona State University, Tempe, PO-Box 878809, AZ 85287, USA

## Abstract

**Motivation:**

In the last years more than 20 vertebrate genomes have been sequenced, and the rate at which genomic DNA information becomes available is rapidly accelerating. Gene duplication and gene loss events inherently limit the accuracy of orthology detection based on sequence similarity alone. Fully automated methods for orthology annotation do exist but often fail to identify individual members in cases of large gene families, or to distinguish missing data from traceable gene losses. This situation can be improved in many cases by including conserved synteny information.

**Results:**

Here we present the SynBlast pipeline that is designed to construct and evaluate local synteny information. SynBlast uses the genomic region around a focal reference gene to retrieve candidates for homologous regions from a collection of target genomes and ranks them in accord with the available evidence for homology. The pipeline is intended as a tool to aid high quality manual annotation in particular in those cases where automatic procedures fail. We demonstrate how SynBlast is applied to retrieving orthologous and paralogous clusters using the vertebrate *Hox *and *ParaHox *clusters as examples.

**Software:**

The SynBlast package written in Perl is available under the GNU General Public License at .

## Background

*Conserved synteny *is the (local) maintenance of gene content and order in certain chromosomal regions of related species. Several studies on chromosome evolution [1-5] demonstrated that conserved synteny exists not only between closely-related species but also over very long evolutionary timescales. Long-range conserved synteny is a particularly frequent feature around developmentally important genes [5], demonstrating that rearrangements are not an unbiased random process in genome evolution.

Conserved synteny is, however, not only of interest as a phenomenon in genome evolution, but provides valuable practical information for the analysis of families of homologous genes. It is a long-standing problem in comparative genomics to identify orthologs, i.e. pairs of genes from two organisms that are separated from each other by a speciation event. In general, the task to distinguish true orthologs from paralogs cannot be solved based on pairwise comparisons. Gene loss, differences in evolutionary rates [6], and convergent evolution often distort the sequence similarities to an extent that makes it impossible to determine orthology from the gene tree. Genomic linkage with genes whose orthology relationships are clearer (i.e. which are more conserved across species and have fewer in-species paralogs) than others can be exploited because linked genes likely share their duplication history. Local/tandem duplications place new copies into a new genomic context, large-scale duplications coordinately duplicate the genomic context and gene loss becomes obvious if it leaves large parts of the genomic context intact while erasing the gene of interest. Therefore, conserved synteny information may demonstrate the loss of a particular copy of a gene and hence put a restriction on which extant gene copies are potential orthologs. If the genomic context of duplicated genes has sufficiently diverged prior to a speciation event, synteny can even provide direct evidence for or against orthology.

There are three basic approaches towards automated orthology identification.

1. Similarity-based clustering methods. This group includes the popular reciprocal pairwise best hit approach and refinements (such as Inparanoid[7-9]), as well as more sophisticated methods that initially represent homology as many-to-many relations. In [10], for instance, the "homology graph" graph of initial blast hits is refined by iteratively removing sub-optimal edges.

2. Phylogenomics-based methods (such as the tree-based Ensembl Compara[11] pipeline). These approaches first cluster homologous genes, then construct a gene phylogeny, attempt to reconcile it with a prescribed species tree and use the resulting mapping between gene-tree and species tree to assign orthology and paralogy relations. An alternative use of phylogenetic information is made by PhyOP, synonymous rate estimates to distinguish between orthologous and paralogous segments in closely related genomes [12].

3. Methods utilizing conserved synteny to infer true orthology between relatively recently diverged species. Methods range from whole genome alignments to combinations with similarity- and phylogenomics-based approaches. Examples are the commercial "syntenic-anchor" approach from Celera [13], the former Ensembl Compara pipeline (prior the June 2006 release). Algorithms that are primarily designed to determine syntenic regions and break points between them also fall into this category [14-18].

Despite substantial improvements in this area, the automatically generated results are still far from being perfect, and the annotation provided by databases such as Ensembl Compara[11] or OrthoDB[19] are neither sufficiently complete nor sufficiently accurate for many applications. For instance, an in-depth study of lineage-specific differences in a family of transcription factors requires not only the complete complement of family members in each species, but also a flawless gene phylogeny (which implies a correct orthology assignment).

The SynBlast tool is designed to assist the manual curation of such data and to focus on individual loci of interest. In contrast to most approaches to genome-wide orthology annotation, it does not operate on pre-determined gene (proteome) sets but it searches the nucleotide sequence of the entire target genome. Hence it does not exclude possible homologs only because they are missing from annotation tracks. Instead of attempting to automatically extract an assignment of orthologs and paralogs, SynBlast instead provides the user with detailed information on all plausible homologs and their genomic context. To this end, web-based graphical overviews that can easily be compared with one another are generated. While heuristic rules are employed by the software to propose a plausible rank-ordering of the homologs with the aim of determining the correct orthologs, its primary purpose is to present conflicting information to the researcher in such way that it facilitates the decision of a human curator.

## Results

### Algorithms and Implementation

#### Overview of the SynBlast pipeline

SynBlast is a "semi-automatic" pipeline that is implemented as a suite of Perl scripts (SynBlast package). In order to allow automatic retrieval of proteins from syntenic regions and comparison of assignments with existing annotations the Ensembl system and databases [11] were chosen as standard reference sources. Therefore, the pipeline scripts make use of the Ensembl Perl API to retrieve reference annotation and sequences from the Ensembl Core databases as well as homology annotations (for comparison only) from the Ensembl Compara database.

**Figure 1:**
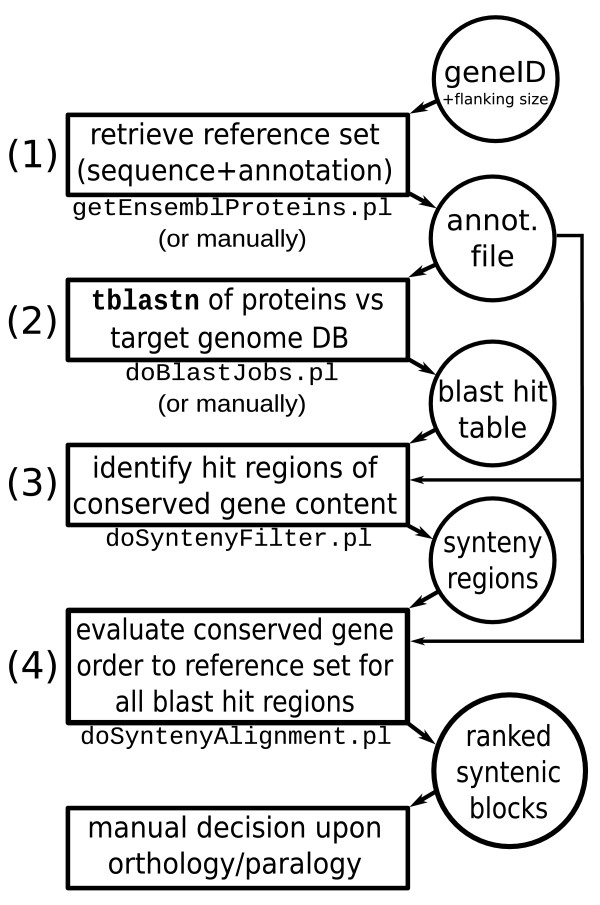
**SynBlast pipeline steps**. (1) A focal protein coding gene of interest and its surrounding genes (within a certain flanking size) are selected as reference set. Protein sequences and genomic positional information are either compiled manually or retrieved from Ensembl using the tool getEnsemblProteins.pl. (2) tblastn searches of all reference proteins are performed against selected target genome databases. (3) The resulting blast hit tables are scanned for regions of possibly conserved gene content. These regions are stored in separate blast hit tables. (4) The resulting sets of possibly syntenically conserved blast hits are evaluated based on their sum of blast bit-scores obtained by means of a gene loci order alignment to the reference set. The final decision on orthology or paralogy of ranked syntenic blocks is left to the user.

The workflow is summarized in Figure 1. It starts from a focal protein coding gene of interest whose homologs are to be detected.

Step 1, in order to include synteny information, the adjacent protein coding genes within a certain genomic distance (flanking size, e.g. 1 Mb up- and downstream) are added to the reference set. The system requires both the sequences and positional information (orientation and (relative) start and end positions) of the reference genes. This information can be provided manually by the user in form of a text file containing tab-separated entries of gene identifiers and their genomic coordinates. Details are given in the SynBlast Tutorial, which is included in the Online Supplemental Material [20]. Alternatively, the corresponding files can be generated using the getEnsemblProteins.pl script which retrieves sequences and annotation information from Ensembl databases.

Step 2 consists of translated-blast searches using all reference proteins as queries on a selection of genome databases as targets. Resulting blast hits are expected to be in tabular (NCBI BLAST) format for further processing. Again this step can be performed manually using any program that creates blast-like tabular output, including NCBI BLAST[21], WU-BLAST (), or BLAT[22]. When the genome data is available locally in NCBI BLAST format, the script doBlastJobs.pl automatizes this step using local NCBI BLAST. It is needed to include the reference species as target genome as well in order to enable the subsequent normalization of blast scores and to detect possible paralogous clusters that the user should be aware of when interpreting final pipeline results. Those paralogous regions of the reference set should be used as reference in a subsequent pipeline run as well to avoid false positive orthology assignments.

In step 3, we search for potential regions of conserved synteny (syntenic target blocks). To this end we collect blast hits that are located within regions of limited size on the target genome. The purpose of this filtering step is to extract candidate subsets of blast hits (or HSPs, high scoring pairs) that can be treated separately in the following. At this stage we do not consider gene order, but gene content information, i.e. a user-specified number of query-specific hits must be contained at minimum in each candidate subset. The procedure is implemented in the script doSyntenyFilter.pl, and is described in detail in the following section.

**Figure 2:**
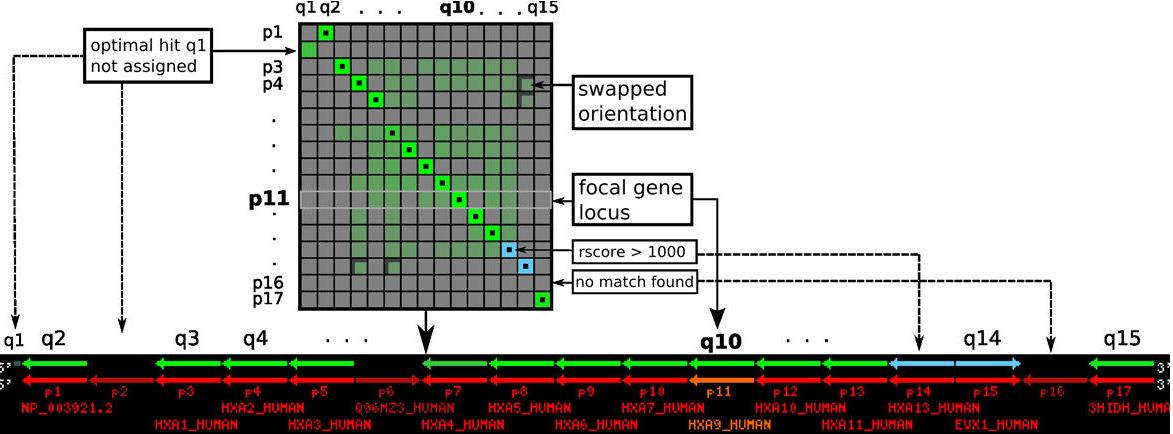
**Dotplot graphic and detailed alignment graphic**. The dotplot (top) visualizes the content of the alignment scoring matrix used to calculate the global alignment with free endgaps where the reference loci (p) and target loci (q) are arranged in rows and columns, respectively. Brightness of green color indicates the relative score value (rscore) of sequence similarity for a particular locus match. The dotted squares correspond to the optimal alignment path, which is shown in the alignment drawing (black box). Swapped orientation of a single target locus (w.r.t. a matching reference locus and the cluster orientation) is indicated by shaded boxes and opposite arrow orientation in the dotplot and alignment drawing, respectively. (Note that in this example the alignment path contains only aligned loci which share the same orientation.) The focal reference gene is highlighted by a light frame and orange color in the dotplot and alignment drawing, respectively.

In step 4, all detected candidate regions of conserved synteny from step 3 are evaluated w.r.t. their agreement with the reference gene order. The technical details of the doSyntenyAlignment.pl procedure are described below. The candidate syntenic regions are sorted according to a scoring scheme that combines sequence similarity, synteny information, and orthology versus paralogy information. The results are presented as HTML files in a web browser together with graphical representations of gene order alignment matrices and paths. Graphics such as those shown in Figure 2 allow the user to readily identify small-scale rearrangements such as translocations or inversions.

#### Extraction of syntenic target blocks – doSyntenyFilter.pl

A region of one of the target genomes is considered as a candidate for a syntenic target block if it contains blast hits from at least *N *different proteins of the query set within an interval of length at most *L*. The parameters *N *and *L *are set by the user, both either directly or indirectly (relative to the reference set). They reflect the expected rate of gene loss and the expected structural similarity. The maximal regions of contiguous blast hits fulfilling these criteria are extracted separately for each target sequence. Depending on the status of the genome assembly, the sequence can be on a chromosome, a scaffold, or a contig. Small scaffolds or contigs pose a problem for this step as a target block syntenic to the query region may be mapped to several different scaffolds. In this case, SynBlast reports two or more separate syntenic regions and/or misses parts of the regions if less than *N *query proteins map to some of the scaffold regions. Note that for some genomes allelic variants are assembled into different scaffolds. SynBlast then reports all these scaffolds and it is left to the user to recognize this.

In its current implementation, the candidate subsets of blast hits are found by a sliding window approach. In addition to the number of query proteins ≥ *N *that have blast hits within a sequence window of size ≤ *L *also the sum of all maximal HSP bit-scores for these proteins is recorded. This yields a convenient measure to prefer the higher-scoring subsets when there are overlapping intervals (in particular if *L *and *N *are too small). We currently use a greedy approach that selects a specified number of target block intervals in decreasing order of the score sum and skips all intervals overlapping a previously selected interval by more than a specified threshold. For a detailed description of the various effects following changes to the parameter settings of *L *and *N *we refer to the SynBlast tutorial, which is provided as part of the Supplemental Material [20].

#### Evaluation of syntenic target blocks via gene order alignment – doSyntenyAlignment.pl

**Figure 3:**
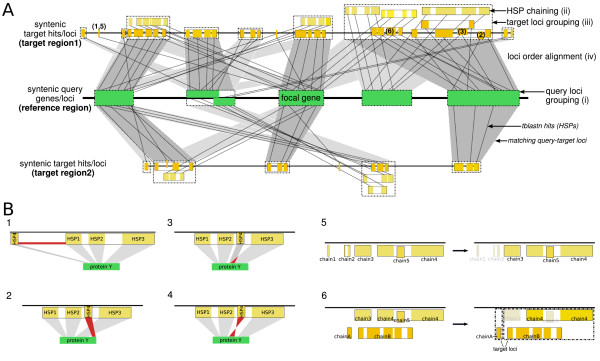
**Evaluation of syntenic blocks**. Panel **(A) **summarizes the mappings from query to target, panel **(B) **elaborates on particular cases. The query region (panel **(A)**, middle) contains a sequence of syntenic query loci (green), each representing one or more possibly overlapping query proteins (i). Each candidate target region in the genomes of interest (panel **(A) **above and below the query locus), is identified by a set of blast hits, HSPs, (yellow). For each region, the following steps are performed: First, the set of query-specific HSPs is chained (ii), resulting in one or more HSP chains that represent approximate protein models (small boxes). Filtering rules are applied that exclude individual HSPs from a chain for one of the following four reasons: *(1) *if the resulting chain exceeds the prescribed size limit for a locus (**B1**) [default: twice the length of the query locus]; *(2) *if it is inconsistent with a co-linear ordering of other HSPs in the chain (**B2**); *(3) *if it overlaps with another query interval by more than a specified threshold (**B3**) [default: 30aa]; and *(4) *if it lies on the opposite strand (**B4**). Chains of HSPs are excluded if they score below a threshold bit-score [default: 50] after filtering (**B5**). The retained HSP chains are grouped (iii) into target loci (big open boxes) that contain all HSP chains (irrespective of their orientation) with overlapping target intervals. For each target locus, only the highest scoring chain for each query protein is kept (**B6**). This results in a sequence of non-overlapping target loci (recall that one locus might represent one or more proteins) that can be aligned (iv) with the sequence of query loci in a gene order alignment (gray shading, optimal assignments are shown by darker shading). The score of this alignment is then used to rank the region relative to other syntenic target regions.

In the fourth step of the pipeline, each of the syntenic target blocks (subsets of blast hits), resulting from step 3, is analyzed separately in comparison to the query region. This part of the pipeline consists of several sub-components, which we discuss separately, see also Figure 3.

(i) The set of reference proteins is linearly ordered (by start/end or mean positions) into so-called "query loci". If the query region contains overlapping proteins, these are combined into a single query locus. Thus, a query locus may comprise more than one query protein (an example is the second query locus in Figure 3A).

(ii) For each query protein we chain all corresponding HSPs into models of target loci. Each HSP consists of an interval *α *= [*a*', *a*"] on the query sequence and a corresponding interval *β *= [*b*', *b*"] on the target sequence. These intervals are linearly ordered for both query and target based on their coordinates.

Intervals on the two strands of a target are treated separately. We furthermore take into account that HSPs must not be too far away from each other, i.e. the maximal genomic extension is restricted to either an absolute or query gene dependent length (locus size limit; see also Figure 3-B1). Again, groups of intervals that are too far apart from each other are treated separately.

For each group of HSPs with common orientation we compute an optimal "alignment" of these lists of intervals using a variant of the Needleman-Wunsch algorithm, similar to Ref. [23]. The query and target intervals of the HSPs, respectively, are considered as characters within the alignment in which a match occurs if both intervals belong to the same HSP. The score of the match is its bit-score. Pairs of (query/target) intervals that do not correspond to the same HSP are considered as mismatches with score -∞. Small negative scores are given to insertions and deletions, endgaps are treated as free. The resulting alignment then defines a consistent chaining of collinear HSPs, here called an "HSP chain" for short. It represents an overall hit for the respective query protein (approximate gene model) with a group score equal to the sum of bit-scores of all HSPs of the chain. A score threshold can be specified to eliminate spurious hits, which otherwise might lead to incorrect groupings of adjacent loci in the next sub step, see also Figure 3-B5. Each HSP chain is furthermore characterized by a start, end, and mean position.

(iii) As in sub-step (i), we now define a linear order of HSP chains by grouping them according to start/end or mean positions into so-called target loci, see Figure 3. If there are HSP chains that reside within the same target locus, (i.e. have overlapping intervals with some HSP chain) while representing hits for the same query protein, only the top-scoring chain is kept, see Figure 3-B6. Thus, we finally end up with linearly ordered blocks of target loci each containing one or more (overlapping) query-specific HSP chains.

(iv) As in sub-step (ii), we use a variant of the Needleman-Wunsch algorithm to obtain a maximum weight sequence of collinear pairs of query and target loci. As before, mismatches are prohibited. Matches are scored as the arithmetic mean of the scores of all matching individual query proteins that belong to the same query locus. Formally, for each pair (*q*, *t*) of query and target locus let *ν*_*qt *_be the number of matching query proteins between the pair of loci. The corresponding similarity score is

(1) $$S(q,t)=\{\begin{array}{ll} \frac{1}{\nu_{qt}}\sum_{s\in q}b(s,t_{s}), & \text{if }\nu_{qt}\geq 1\\ -\infty & \text{otherwise} \end{array}$$

where *b*(*s*, *t*_*s*_) is the bit-score of query protein s with its match *t*_*s *_on the target genome as determined in sub-step (ii). In contrast to step (ii), we do not exclude matches between items of different orientation. Instead, we use only a fraction of the score *b*(*s*, *t*_*s*_) (adjustable parameter) to penalize those matches. Thus, swapped orientation of a target locus w.r.t. the orientation of its matching query locus within the reference set is generally allowed, but the match score of such a locus is reduced to a user-defined fraction (e.g. 90%). This parameter can also be set to 0, in which case matches with reversed direction are considered as not informative at all. The gene order alignment score is consequently calculated for both orientations of the target block relative to the reference set, and only the higher-scoring alignment is retained in subsequent steps.

In addition to this absolute scoring we also compute relative weights *b*(*s*, *t*_*s*_)/*b*(*s*, *q*_*s*_) where the absolute bit-score is scaled by the score obtained by matching the query protein *s *back to its genomic locus *q*_*s *_within the reference genome. The value *b*(*s*, *q*_*s*_) is a good approximation for the maximal tblastn score of a given query protein. The relative score is then used as match score during the gene order alignment. This ensures that the matches to each reference locus are scored relative to the information content of the locus.

Since match scores are defined directly between loci we can conveniently combine the visualization of the alignment path and the scoring matrix, see Figure 2.

(v) Finally, all evaluated target regions are compiled in a ranked list in browsable HTML format including graphical overviews of loci scoring matrices (dotplot) and alignments as well as an alignment table displaying additional information for assigned loci. Swapped orientation of a single target locus is indicated in the dotplot by a shaded dot. In the alignment graphics, an arrow with reversed orientation w.r.t. the arrow of its assigned query locus is used.

The ranking can be based on the gene order alignment score (roughly the sum of (weighted) bit-scores for assigned target loci) or the *(log)RatioSum *score, which is calculated as the sum of (the logarithms of) intra-inter-score ratios of assigned target loci. This score ratio, which is described in detail in the "Methods" section, measures the ambiguity of orthology between two loci based on the existence of close paralogs within the reference. It has proven useful in the process of identifying true orthologs. In combination with the gene order alignment score this score makes it easier to distinguish between putative orthologous and paralogous hits or clusters.

If reference and genome data are taken from Ensembl databases, SynBlast optionally retrieves the EnsemblCompara homology annotations and the Ensembl Core protein coding gene annotations overlapping the target locus interval of the matching HSP chain identified by SynBlast, for comparison.

### Applications

As a real-life test of SynBlast, we consider here the genomic clusters of vertebrate *Hox *and *ParaHox *genes. These genes code for homeodomain transcription factors that regulate the anterior/posterior patterning in most bilaterian animals [24,25]. *Hox *and *ParaHox *genes arose early in metazoan history from a single ancestral "*UrHox *gene" [26]. After a few tandem duplication events, a large scale duplication lead to ancestral *Hox *and *ParaHox *clusters. While the ancestral *ParaHox *cluster remained largely unchanged, the evolution of its *Hox *counterpart was dominated by a series of tandem duplications. As a consequence, most bilaterians share at least eight distinct paralogous groups (8 in arthropods, and 13 or 14 in chordates) which retained high sequence similarity at the homeobox. Both *Hox *and *ParaHox *genes are usually organized in tightly linked clusters [27], with syntenic conservation extending even beyond the core clusters themselves. For instance, an additional homeobox gene, *Evx*, located at the 5' end of the *Hox *cluster can be seen as part of an extended *Hox *cluster, see [28] for more details.

The modern vertebrate genome arose from an ancestral chordate by means of two rounds of whole genome duplication [29,30]. Teleost fishes have undergone an additional round of genome duplication [31,32].

Substantial loss of duplicated genes was the consequence of these duplication events. In the case of *Hox *clusters there is little doubt about the orthology relationships among the *Hox *genes of tetrapoda. In teleost fishes, however, the relationships of the duplicated *Hox *clusters between zebrafish and fugu have long been controversial, see [33] for a discussion, and have only recently been resolved using a dense taxon sampling [30]. It is well known that the relative order and orientation of *Hox *genes in their clusters have been highly conserved in vertebrate evolution, albeit there is substantial gene loss. The *Hox *clusters thus are an excellent test case to demonstrate the gene order alignment functionality of SynBlast.

#### Vertebrate *Hox *clusters

**Figure 4:**
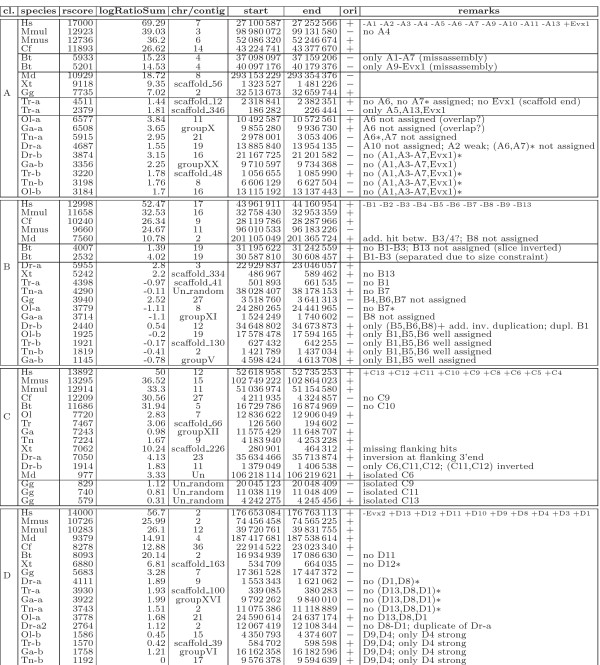
**Overview on pipeline results for vertebrate Hox clusters**. SynBlast results and manually extracted orthologous cluster positions and identities for selected vertebrate species are listed. Unless otherwise indicated, positions correspond to assigned blast hits' intervals from *Hox1 *to *Hox13*/*Evx *hits in gene order alignment. Cluster orientation is w.r.t. the human reference clusters, which are HOXA9_ENSG00000078399_5e5; HOXB9_ENSG00000170689_2e5; HOXC9_ENSG00000180806_3e5; HOXD9_ENSG00000128709_5e5. Unassigned loci from the reference may be due to overlaps of chained HSPs. A '*' indicates loci that are absent in agreement with the literature [45]. Data for Ensembl release 42 (Dec 2006).

We used the four human *Hox *clusters as reference and searched the vertebrate target species with SynBlast. We consider here a diverse set of vertebrate genomes which contains both tetrapods (with 4 paralogous *Hox *clusters) and teleosts (with 8 paralogons). The cluster locations, gene inventories, and SynBlast scores are listed in Figure 4. In case of genomes with complete assemblies, the correct assignment of cluster orthology and the correct assignment of *Hox *gene identity is straightforward by visual inspection of the SynBlast cluster alignments, see Table 1 for an example. Here, both the gene order alignment score and the *logRatioSum *score is suitable to assign cluster identity to the target loci in the zebrafish genome. However, the *logRatioSum *score clearly out-performs the gene order alignment score in case of the *Danio Bb *cluster. In combination, the two scores provide the best means to rank orthologous loci at the top. The zebrafish Zv7 assembly contains two inparalog copies *DrCa1 *and *DrCa2 *of the zebrafish *HoxCa *cluster. This is, however, certainly an assembly artifact and contradicts all of the existing literature, see e.g. [34] and the references therein. SynBlast correctly retrieves both copies with comparable scores.

**Table 1:** SynBlast results for *Danio rerio*, Ensembl release 46 (Zv7), with the human *Hox *clusters as query.

Reference	*DrAa * chr.19 10.5 M	*DrAb * chr.16 16 M	*DrBa * chr.3 23 M	*DrBb * chr.12 26.5 M	*DrCa1 * chr.23 33.7 M	*DrCa2 * chr.23 35.2 M	*DrCb * chr.11 0.6 M	*DrDa * chr.9 2 M
*HsA*	**2.58**	**3.29**	-0.76	-0.39	-1.98	-1.98	-0.4	-0.74
*HOXA9*	**5581**	**4073**	4690	2119	3322	3318	1490	3269
chr.7	**2/1**	**1/3**	7/2	3/7	9/4	10/5	4/8	6/6
								
*HsB*	-1.14	-0.01	**3.13**	**0.42**	-1.66	-1.69	-0.64	-0.48
*HOXB9*	2702	1342	**6201**	**1647**	3008	2982	1003	1678
chr.17	6/4	3/7	**1/1**	**2/6**	7/2	8/3	5/8	4/5
								
*HsC*	-0.89	-1.51	-0.26	-0.34	**6.44**	**7.58**	**0.22**	-2.51
*HOXC9*	2361	2323	3108	1346	**8687**	**6537**	**4150**	3798
chr.12	7/6	8/7	5/5	6/8	**2/1**	**1/2**	**3/3**	9/4
								
*HsD*	-0.92	-0.63	-0.85	-0.6	-0.41	-0.41	-0.71	**1.76**
*HOXD9*	2799	1811	2660	871	3017	3013	1303	**4326**
chr.2	8/4	5/6	7/5	4/8	3/2	2/3	6/7	**1/1**

The *logRatioSum *score, the gene order alignment score, and the corresponding ranks are given. Putative orthologs are depicted in bold. In combination, the two scores provide the best means to rank orthologous loci at the top. In most cases, identification of orthologous regions is unambiguous. For completeness we list both copies of the artifactually duplicated DrCa cluster of chr.23.

In the case of incomplete assemblies, only partial clusters can be obtained. For instance, individual *Hox *loci of oppossum and chicken are located on small separate scaffolds. For the duplicated genomes of the five teleosts in our data set, we obtained all 7 *Hox *genes-containing paralogous clusters in agreement with the literature, see [33,35-38]. Since our query consisted of the *Hox *cluster only, we could of course not retrieve the 8th zebrafish paralogon, which is completely devoid of homeobox genes [39].

Several artifacts of preliminary genome assemblies further complicate the analysis: In the fugu *HoxAa *cluster we readily detected the artifactual breakage of the cluster into two fragments on scaf.12 and scaf.346. In the older Zv6 assembly of the zebrafish genome, some *Hox *clusters contained local rearrangements and obviously duplicated gene loci, in particular the *HoxBb *and *HoxCb *clusters. Most of these problems have been resolved in the most recent Zv7 assembly, while the *Ca *artifact has been newly introduced. Table 2 summarizes the discrepancies of the Ensembl Compara annotation for the orthology assignments obtained using SynBlast, the latter conforming to the recent literature [33].

**Table 2:** Incomplete and erroneous Ensembl Compara orthology annotations for vertebrate *Hox *cluster loci.

Ref. cluster	Genes			Clusters	
		
	Δ	*β*	*n*	*M*	*K*
*HoxA*	11	2	148	5	16
*HoxB*	25	2	118	12	16
*HoxC*	11	2	88	6	10
*HoxD*	20	7	113	13	16

We list the total number *n *of *Hox *and *Evx *genes in the dataset; the number Δ of *Hox *and *Evx *gene orthology assignments (see Figure 4 that are well-supported (query coverage > 30%) by SynBlast but that are missing in the Compara annotation; the number *β *of well-supported assignments with incorrect annotations in Compara (paralog). We furthermore list the number *M *of the *K Hox *clusters in the dataset which contain apparently missing and/or erroneous Compara annotations. All data refer to Ensembl release 42 (Dec 2006).

The very well-understood example of the *Hox *gene cluster demonstrates that the true orthology and paralogy relationships can be determined rather quickly and easily by means of a manual analysis with the assistance of SynBlast. Automatic orthology annotation pipelines, on the other hand, still produce unsatisfactory results despite recent progress.

#### Teleost ParaHox clusters

The *ParaHox *clusters of teleost fishes have long been used to contradict the whole genome duplication scenario because of a mainly unduplicated repertoire of *ParaHox *genes compared to other vertebrates. Even after the teleost-specific genome duplication had been broadly accepted, the small number of *ParaHox *genes in each cluster and the large amount of gene loss at this locus complicated attempts to decipher their duplication history. Knowledge about the number of paralogous *Cdx *genes and their assignment to paralogous groups is a good starting point for such a reconstruction. Two studies based on publicly available genome sequences arrived at different scenarios for the history of this particular *ParaHox *gene in teleost fishes [40,41]. While Prohaska et al. [40] proposed the existence of a *Cdx2 *gene copy (at least for fugu and tetraodon), Mulley et al. [41] concluded that both copies of *Cdx2 *were lost and suggested that this loss was compensated by two copies of *Cdx1*. A more recent analysis that uses additional sequence data [42] settles the discrepancy in favor of [41], supporting the retention of two *Cdx1 *genes in cichlids. The analysis of [42] in part excludes zebrafish because of problems with the available genome assemblies. Here we demonstrate how SynBlast can be used to facilitate retrieval of candidate *Cdx *loci and cluster assignments in the zebrafish genome.

**Figure 5:**
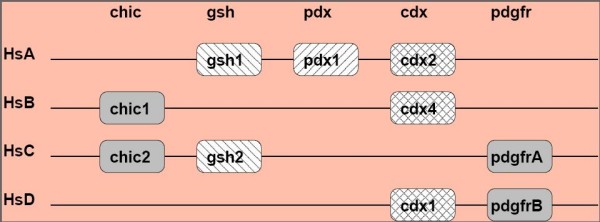
**Schematic representation of genes flanking the Cdx gene locus in the human ParaHox clusters**. Only linked genes relevant for the interpretation of the *SynBlast *output are shown.

In an intact *ParaHox *cluster, the *Cdx *gene is flanked by two *ParaHox *genes, i.e. *Gsh *and *Pdx*, and a number of genes of other gene families. According to [42], the ancestral gnathostome *ParaHox *genes are organized in four clusters, designated A, B, C, and D in analogy to the *Hox *clusters (see Figure 5). The *Cdx *gene of the C cluster has been lost soon after the 1R/2R duplications [41]. No organism with a fourth *Cdx *paralog resulting from this duplication event has yet been found. Therefore, only three of the four ancestral clusters each retained a *Cdx *gene: *Cdx2 *(cluster A), *Cdx4 *(cluster B), and *Cdx1 *(cluster D). As a consequence of the teleost genome duplication we would expect to find 8 *ParaHox *clusters, two A clusters (A1, A2), two B clusters (B1, B2) etc. and up to 6 *Cdx *genes in the 6 clusters A1/2, B1/2, and D1/2. We start our SynBlast search in the zebrafish genome with the four human *ParaHox *cluster regions.

Table 3 shows the scores for all pairs of the four human query loci with the 11 high-scoring target loci of zebrafish. The assignment of true orthologs is more challenging than in the case of *Hox *clusters but still revealing.

**Table 3:** SynBlast results for *Danio rerio*, Ensembl release 46 (Aug 2007), Zv7 assembly with the human *ParaHox *clusters as query.

Reference	*DrA1 * chr.24 20 M *pdx1*	*DrA2 * chr.5 60 M *gsh1*	*DrB * chr.7 50 M	*DrB1 * chr.14 37 M *cdx4*	*DrC1 * chr.20 20 M *gsh2*	*DrC2 * chr.1 10 M	*DrD1*? chr.14 53 M	*DrD1*? chr.14 22 M	*DrD1 * chr.14 25 M *cdx1a*	*DrD2 * chr.21 43 M *CDX1*	*DrD2*? chr.21 36 M
*HsA/C1*	**2.49**	**0.25**	0.52	0.05	-0.65	-	-	-	1.3	-	(0.13)
*CDX2*	**8343**	**2291**	1605	1537	1922	-	-	-	1757	-	(1112)
chr.13	**2/1**	**7/2**	5/5	9/6	40/3	-	-	-	3/4	-	(8/8)
											
*HsB/C2*	(-0.41)	(-0.7)	**2.53**	**0.47**	(-0.04)	-0.02	-	-	-0.75	-0.14	-
*CDX4*	(237)	(139)	**2934**	**1192**	(114)	669	-	-	543	183	-
chr.X	(19/25)	(31/38)	**1/1**	**3/2**	(7/41)	5/7	-	-	34/10	12/32	-
											
*HsC/C3*	-1.47	-1.56	-0.33	-2.52	**3.23**	**0.91**	-	-	-0.46	-	(-0.37)
*GSH2*	1478	507	1424	1595	**4756**	**1880**	-	-	469	-	(488)
chr.4	54/4	55/7	19/5	56/3	**1/1**	**3/2**	-	-	26/9	-	(23/8)
											
*HsD/C4*	-0.6	(-2.77)	(-3.57)	-3.44	-3.31	(-0.06)	**0.79**	**0.03**	**-0.41**	**0.18**	**5.27**
*CDX1*	439	(254)	(2238)	953	940	(991)	**1796**	**1514**	**846**	**1107**	**2550**
chr.5	13/41	(24/57)	(42/2)	41/12	37/13	(7/11)	**2/4**	**6/5**	**11/16**	**4/9**	**1/1**

The *logRatioSum *score, the gene order alignment score, and the corresponding ranks are given. Putative orthologs are depicted in bold. In combination, the two scores provide the best means to rank orthologous loci at the top. Numbers in parentheses indicate that the target region (column) is only approximately matched, and/or that only a single query gene was found. See text for more detail.

**Figure 6:**
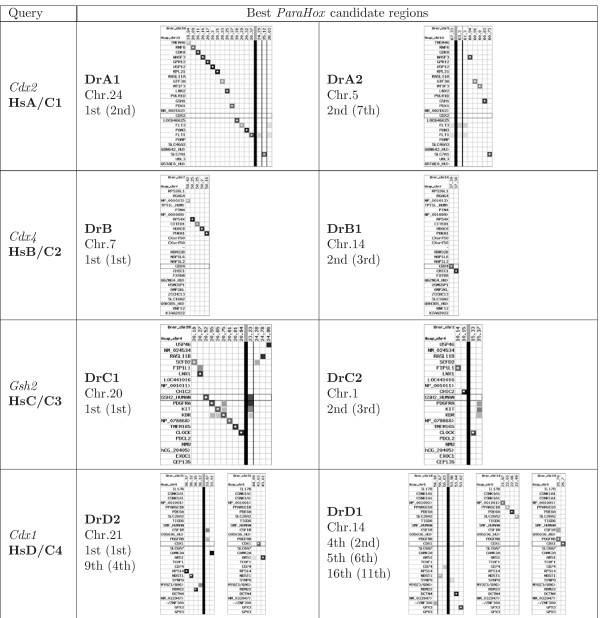
**ParaHox example application**. SynBlast was used to determine the four pairs of paralogous regions generated by the fish-specific genome duplication from the four gnathostome *ParaHox *regions. We show alignment dot-plots for the high-ranking hits (according to the gene order alignment score and *logRatioSum *score (in brackets)) of the four query regions against the zebrafish genome (Zv7, Ensembl release 46, Aug 2007). Parameters for the synteny filtering step were *N *= 1, *L *= 2. See text for more details.

One copy of *ParaHoxA *retained 13 of 24 genes flanking the *Cdx2 *locus even though *Cdx2 *itself was obviously lost. This is a case where gene loss can reliably be distinguished from missing data based on well-conserved synteny information (see Figure 6). The second copy retained only 5 of the 24 flanking genes. In line with the analysis of [41,42], we observe that the *Cdx2 *gene has been lost from both copies. We also observe that one of the two *ParaHoxA *contains the only copy of *Gsh1 *which is located at **DrA2 **(Chr.5), while the only copy of *Pdx1 *is located at **DrA1 **(Chr.24). Note that this information independently confirms the assignment of the two zebrafish *ParaHoxA *paralogs to the ancestral A cluster. SynBlast reports additional syntenic regions in the zebrafish genome that contain homologs of some of the genes of the *HsA *query. These are located on chromosomes 7, 14, 20, and 21, and can be assumed to be orthologs of the *ParaHox *B, C, and D clusters. In order to confirm this assumption, we also consider the remaining three human *ParaHox *regions as queries.

The query with human *ParaHoxB *yields only poorly conserved synteny information. This can be due to the reorganization of this locus when it got translocated to the mammalian sex chromosome X, see [40,43] for details. Nevertheless, we obtain sufficient information from the linkage of the *Cdx *loci with *chic1 *to see that zebrafish has one *Cdx4 *locus, **DrB1 **(Chr.14). With the human *ParaHoxC *cluster, which contains the *Gsh-2 *gene as a query, two paralogous regions in the zebrafish genome can be identified. **DrC1 **on Chr.20 containing the only surviving copy of *Gsh2*, while a putative **DrC2 **locus on Chr.1 contains three high-scoring reference loci (*fip1l1*, *chic2*, and *clock*) and both neighbors of *Gsh2*, i.e. *chic2 *and *pdgfrA*, but is devoid of homeobox genes.

Note that "empty" parahox clusters are not exceptional. Teleost fishes have also lost all homeobox genes in one of the *HoxD *paralogs (zebrafish, [39]) or one of the *HoxC *paralogs (pufferfish, [40]), respectively. Loss of a gene of interest can nevertheless be identified due to the retention of neighboring genes given sufficient conserved synteny.

The assignment of orthologs to cluster *HsParaHoxD *is difficult. Conserved synteny information is relatively rare and only locally given, i.e. the orthologous hits for those query regions are scattered more or less across the target chromosome or even genome, which is probably due to extensive rearrangements. Nevertheless, one *Cdx *locus is linked to *pdgfrB*, a D cluster gene. SynBlast detects multiple fragments that map to two distinct zebrafish chromosomes. A plausible hypothesis is to interpret the three hits of Chr.14 at 22 M, 25 M, and 53 M as remnants of one dissolving cluster **DrD1**, while the two fragments of Chr.21 at 36 M and 43 M constitute the other **DrD2 **paralog.

In summary, we have located the three retained *Cdx *genes in the highly fragmented zebrafish genome assembly, and we conclude that three *Cdx *genes were lost in the aftermath of the fish-specific genome duplication. Due to synteny information, the three *Cdx *genes can unambiguously be assigned to the paralog groups *Cdx4 *(one copy, B cluster) and *Cdx1 *(two copies, D clusters).

#### Further Examples

In addition to the difficult *Hox *and *Parahox *loci we have investigated several examples of human loci with extensive synteny in other vertebrates, some of which are included in the Online Supplemental Material for comparison. In these rather straightforward cases we encountered a rather common annotation problem. Homology-based protein annotation sometimes produces two (or even more) disconnected annotated fragments, in particular when evidence from different sources is used. Since these fragments are not recognized as parts of the same protein, they are subsequently interpreted as distinct homologs of the same protein, resulting in an erroneous "within-species-paralog" assignment. We observed that SynBlast correctly recognizes such disconnected fragments as belonging to the same query item in the HSP chaining step, and hence avoids these spurious "paralogs".

## Discussion and Conclusion

The SynBlast tool was developed to assist in the interactive preparation of high-quality orthology annotations. It uses synteny in addition to sequence similarity. A major difference to most other tools is that it does not operate on a "proteome set". Instead, it uses tblastn and a two-level alignment procedure to retrieve the homologs of a set of reference proteins. As a consequence, it is independent of gene predictions and annotations of the target genomes. Known or predicted protein sequences are required only for the query genome. This avoids in particular many of the problems with misannotations in the target genome that may confuse automatic pipelines.

A major advantage of the synteny-based approach is that we also find fairly diverged homologs in a conserved context that would otherwise be discarded due to insufficient sequence similarity, see also [44]. This allows the user to find supporting information for highly diverged genes or gene loss and to distinguish it from the failure to detect sequence similarity. As a consequence, we find that SynBlast is particularly useful to retrieve homologous regions in the presence of high rates of gene loss, such as after the teleost-specific genome duplication. Syntenic regions are found and gene losses can be identified even when the focal genes are lost from one or more paralogons. As demonstrated in the *ParaHox *cluster example, information on such loci is readily accessible using SynBlast and can be instrumental in deciphering complex duplication/loss scenarios. This is the case in particular when homologous genes that arose through several distinct duplication events are of interest, as in the case of homeobox clusters. Of course, in cases where synteny is not preserved, SynBlast cannot do better than a simple blast search. In such a case, the output of the program at least makes it easy for the user to identify cases of disintegrated synteny. To distinguish orthologous and paralogous regions, SynBlast provides two scoring schemes: one that attempts to evaluate the overall similarity of two syntenic regions (gene order alignment score), and alternatively the relative similarity in comparison to the most similar within-reference paralog (*(log)RatioSum *score). However, the SynBlast system was designed to aid a careful manual evaluation rather than to provide an automatic pipeline. Hence, it produces extensive graphical and tabular output of all regions in the target genomes that are potentially syntenic to the query region in the form of HTML pages, which also integrates the existing Ensembl Compara homology annotation for comparison. This renders the tool most useful when orthology annotation is not obvious and expert knowledge is required to reach a definitive conclusion.

## Methods

The vertebrate genomes were taken from Ensembl (release 42, Dec 2006). In case of the *ParaHox *application and also the *Danio Hox *example the new assembly version for zebrafish (Zv7, Apr 2007 from Ensembl release 46, Aug 2007) was used. The new *Danio *assembly was scanned with local WU-BLAST (tblastn, version 2.0 MP-WashU, 04-May-2006). All other blast searches were performed with local tblastn (blastall version 2.2.15 of the NCBI BLAST suite). Genome databases were used in repeat-masked form, and the minimum *E*-value was set to *E *= 10^-5 ^or *E *= 10^-4^. The maximal size of the target cluster was restricted to twice the size of the reference cluster for all applications (parameter *L*). The number of different proteins to be contained in a valid synteny region at minimum (parameter *N*) was set to 1 (Parahox application) or 4 (Hox application). The cutoff for the HSP chain score was set to 100. The cutoff for the maximal overlap (w.r.t. query coordinates) of neighboring consistent HSPs was set to 40 amino acid positions. The fraction of the score used for matches of loci with different orientation was set to 90 percent while the gap penalties were set to 10 (gap in reference sequence) and 2 (gap in target sequence). All scripts were written in Perl (v5.8.8) and executed on PC hardware running Linux.

The *intra-score *is calculated once for each query protein *s*, and describes the relative difference of the best and the second-best hit onto the reference genome (i.e. for the closest-related paralogs). This is approximated by their bit-score differences, i.e.

(2) $$S_{\text{intra}}(s)=\frac{b(s,q_{s1})-b(s,q_{s2})}{b(s,q_{s1})}$$

where *q*_*s*1 _and *q*_*s*2 _are the two top-scoring target loci (i.e., HSP chains) within the reference genome. The more distant the closest paralogs in the reference, the more reliable is the assignment of orthologs from the target species.

The *inter-score *is calculated for each assigned target locus *t*_*s *_within a target genome, defined as its relative bit-score difference to the best reference hit locus *q*_*s*1_:

(3) $$S_{\text{inter}}(t_{s})=\frac{b(s,q_{s1})-b(s,t_{s})}{b(s,q_{s1})}$$

Hence, the inter-score expresses how "bad" a putative ortholog hit to the target genome is w.r.t. the maximally expected score *b*(*s*, *q*_*s*1_).

The ratio of intra-score and inter-score, *S*_intra_/*S*_inter_, quantifies the quality of an inter-species (potentially orthologous) hit in relation to the similarity between paralogs in the reference genome. Therefore, it serves as a measure for the confidence in the orthology of the query and target locus.

The *(log)RatioSum *score is defined as the sum of the (logarithms of the) intra-inter-score ratios of all target loci assigned within the gene order alignment.

## Availability and requirements

The SynBlast package written in Perl is available under the GNU General Public License at . It requires a Unix-like environment and several add-on perl modules (DBI, GD) installed, as well as an installation of the Ensembl Core and Ensembl Compara APIs of the appropriate release version, see also the SynBlast tutorial [20] for installation issues. A local version of the NCBI BLAST suite, as well as the genome sequence databases of selected target species is needed to generate the genome-wide similarity search results as part of the pipeline.

**Project name: **SynBlast

**Project home page: **

**Operating System: **Unix/GNU Linux

**Programming languages: **Perl, bash

**Other requirements: **several add-on perl modules (DBI, GD), Ensembl Core/Compara API, NCBI BLAST (or similar).

**License: **GNU GPL version 2 or any later version

## Authors' contributions

SJP and PFS designed the study, JL developed the SynBlast pipeline and tool, all authors closely collaborated in preparing the manuscript.
